# Pigment contribution to feather mass depends on melanin form and is restricted to approximately 25%

**DOI:** 10.1098/rsbl.2025.0299

**Published:** 2025-08-20

**Authors:** Ismael Galván, Julene Gómez-Vicioso

**Affiliations:** ^1^ Department of Evolutionary Ecology, Museo Nacional de Ciencias Naturales, CSIC, Madrid, Spain

**Keywords:** bird coloration, evolution of feathers, flight, pigmentation, plumage weight

## Abstract

Feathers are lightweight keratinous structures that have promoted the evolutionary success of birds by facilitating flight. Complex feathers, however, are believed to have evolved in response to visual functions, meaning a relevant role of pigmentation in feather evolution. The most common pigments in birds are melanins, large polymers synthesized at feather follicles, which thus have the potential to contribute significantly to the mass of feathers and compromise their lightweight nature. This contribution has never been quantified. Here, we leveraged a melanin extraction method to measure the mass contribution of different melanin forms to feathers from 19 species of birds, mainly golden eagles *Aquila chrysaetos* and pied flycatchers *Ficedula hypoleuca*. Melanin contribution to feather mass averaged 22.3%, but the contribution of eumelanin, responsible for black/grey/dark brown colour phenotypes, was higher than that of pheomelanin, responsible for lighter phenotypes. Eumelanins with a lower content of indole-carboxylated subunits also contributed more to feather mass. Melanin forms do not exert additive effects and constitute approximately 25% of feather mass regardless of whether the pigment composition is mixed or contains a single form. Our findings introduce a novel metabolic cost for flight associated with different plumage phenotypes that may help understand the evolutionary predictors of bird colour diversity.

## 1. Introduction

Avian evolution is intrinsically linked to the evolution of feathers, tubular epidermal structures consisting of pleated ß-keratin sheets with a hollow central shaft. The evolutionary diversification of birds is highly dependent on the morphological diversity of feathers, which exert, even simultaneously, functions for thermoregulation, visual communication, waterproofing, mechanical protection and flight [1]. Although the most relevant function for the evolutionary success of birds is facilitating sustained flight, the first branching and planar feathers evolved due to the benefits conferred for visual communication, i.e. signalling [2]. For the purpose of visual communication, feathers must normally be coloured, and feathers get coloured most commonly by incorporating melanin pigments in their structure [3]. For the purpose of flight, feathers must get a balance between lightweight and stiffness/strength, which is achieved by structures such as the syncytial cells of the cortex of rachis and barbs, the crossed-fibre architecture of the epicortex and the foam-like structure of the medullary pith [4]. Any material that is added to the keratin structure of feathers, such as pigments, thus has the potential to compromise their lightness. However, the contribution of pigments to the mass of feathers has never been determined, thus leaving it unclear whether the primary function of visual communication can constrain flight to some extent. Quantifying this contribution may lead to identifying potential trade-offs in which pigments are implied, necessary to understand ecological and evolutionary bird colour patterns.

Melanins are polymeric compounds that in birds are synthesized by melanocyte progenitors in the pigmentary units of feather follicles and are transferred to the keratin structures as the feathers grow [5]. Melanins are synthesized as two main chemical forms: eumelanin and pheomelanin, which generate diverse colour plumage phenotypes when deposited on feathers either isolated or mixed at different proportions [6]. Eumelanins are polymers of two indole units, namely 5,6-dihydroxyindole (DHI) and 5,6-dihydroxyindole-2-carboxylic acid (DHICA), while pheomelanins are oligomers of sulfur-containing heterocycles, mainly benzothiazines and benzothiazoles [7]. As melanins are polymers, presenting relatively high molecular masses, these pigments can potentially contribute significantly to the mass of the keratin backbone of feathers.

If eumelanins and pheomelanins contributed differentially to feather mass, that would mean that different colour plumage phenotypes could constrain flight to different extents. Eumelanins are probably larger, thus heavier, than pheomelanins [8], and also form more rigid and compact granules [9]. The subunit composition of eumelanins is also relevant to understanding the contribution of pigments to feather mass, given the different coupling options for polymerization offered by DHI and DHICA [10,11], which result in DHI generating large branched polymers and DHICA generating smaller linear polymers [12].

Here, we experimentally removed melanins from feathers of several species of birds with a diversity of feather melanin compositions, with the aims of quantifying the contribution of melanins to the mass of feathers and testing for differences between melanin forms in such contribution. We predict that the mass of eumelanins should be higher than that of pheomelanins in relation to the mass of feathers, given the larger size of eumelanin polymers, and that eumelanins with a higher relative content of DHI should also contribute more to the mass of feathers, given the larger size of DHI polymers.

## 2. Material and methods

### (a) Species and feather samples

In the analyses, we used feathers pigmented by melanins that have a mixed composition of eumelanin and pheomelanin, as well as feathers with a pure pigment composition of either eumelanin or pheomelanin. We used feathers whose melanin composition had previously been determined by Raman spectroscopy analyses, a methodology that has been validated by chemical degradation studies of feather melanins using high-performance liquid chromatography (HPLC; e.g. [13]), as shown by Galván *et al*. [14]. As models for mixed melanin composition, we mainly used golden eagle *Aquila chrysaetos* feathers [15], but also red-legged partridge *Alectoris rufa* feathers. As a model for pure melanin composition, we mainly used the back plumage of male pied flycatchers *Ficedula hypoleuca*, which is composed of grey and black feathers pigmented only by eumelanin [16], but also used feathers from another seven species of birds pigmented only by eumelanin and nine species pigmented only by pheomelanin. In total, we used feathers from 109 bird specimens belonging to 11 orders and 18 families (53 specimens with mixed pigment composition, 44 specimens only pigmented by eumelanin and 12 specimens only pigmented by pheomelanin). We used white, unpigmented feathers from the belly of five barn owls, *Tyto alba,* as controls. All feathers were body feathers from adult birds, except those from golden eagles, which were juvenile birds.

A sample is here defined as a set of feathers from a single bird specimen, and is also considered the sampling unit. Species and sample sizes, as well as sources of information for pigment composition, are listed in table 1. All feather samples were obtained from wild and captive populations of birds, as well as from museum collections (table 1). Museum collection samples comprised a tight window of specimens’ age ranging from 43 to 78 years (average: 57.7 years), with the exception of a 120-year-old great grey shrike *Lanius excubitor* specimen and a common kingfisher *Alcedo atthis* specimen of unknown age. The total percentage contribution of melanins to the mass of feathers (see below) did not correlate with specimens’ age (*r* = −0.11, *n* = 13, *p* = 0.706), indicating that any potential degradation of melanins in collection specimens did not affect our analyses.

**Table 1.** Species and taxonomic information for each feather sample available for the study. Sample size refers to the number of bird specimens from which feathers were obtained per species. It is indicated whether the specimens were obtained from wild populations, captive populations or museum collections (MNCN: National Museum of Natural Sciences, Madrid, Spain; RM: Icelandic Institute of Natural History, Reykjavík, Iceland). Pigment composition refers to whether only one melanin form or both forms (mixed) are present in the feathers. Information on pigment composition is obtained from published evidence of Raman spectroscopy analyses of feathers, except for superb starling *Lamprotornis superbus* feathers, for which Raman spectra are unpublished (references are indicated by superscripts).

species	order	family	sample size	origin of specimens/samples	pigment composition
*Aquila chrysaetos*	Accipitriformes	Accipitridae	50	wild	mixed^a^
*Ficedula hypoleuca*	Passeriformes	Muscicapidae	37	wild	eumelanin^b^
*Alcedo atthis*	Coraciiformes	Alcedinidae	1	MNCN	pheomelanin^c^
*Alectoris rufa*	Galliformes	Phasianidae	3	captivity	mixed^d^
*Anas crecca*	Anseriformes	Anatidae	1	RM	pheomelanin^e^
*Ardea cinerea*	Pelecaniformes	Ardeidae	1	MNCN	eumelanin^c^
*Corvus corax*	Passeriformes	Corvidae	1	MNCN	eumelanin^c^
*Gavia stellata*	Gaviiformes	Gaviidae	1	RM	pheomelanin^c^
*Lamprotornis superbus*	Passeriformes	Sturnidae	4	wild	pheomelanin^f^
*Lanius excubitor*	Passeriformes	Laniidae	1	MNCN	eumelanin^c^
*Mareca strepera*	Anseriformes	Anatidae	1	RM	pheomelanin^e^
*Motacilla cinerea*	Passeriformes	Motacillidae	1	MNCN	eumelanin^c^
*Phalaropus lobatus*	Charadriiformes	Scolopacidae	1	RM	pheomelanin^e^
*Podiceps auritus*	Podicipediformes	Podicipedidae	1	RM	pheomelanin^e^
*Pterocles orientalis*	Pterocliformes	Pteroclidae	1	MNCN	eumelanin^c^
*Pyrrhula pyrrhula*	Passeriformes	Fringillidae	1	MNCN	eumelanin^c^
*Sitta europaea*	Passeriformes	Sittidae	1	wild	^g^pheomelanin
*Stercorarius skua*	Charadriiformes	Stercorariidae	1	RM	eumelanin^c^
*Upupa epops*	Bucerotiformes	Upupidae	1	MNCN	pheomelanin^c^
*Tyto alba*	Strigiformes	Tytonidae	5	wild	unpigmented

^a^ Galván *et al.* [15].

^b^ Galván [16].

^c^ Galván *et al.* [17].

^d^ Galván *et al.* [14].

^e^ Rodríguez-Martínez *et al.* [18].

^f^ Own unpublished data.

^g^ Galván & Rodríguez-Martínez [19].

### (b) Feather mass and melanin extraction

We weighed empty crystal test tubes to the nearest 0.1 mg using a Precisa LS 220A balance (Precisa Gravimetrics AG, Dietikon, Switzerland). We also weighed a variable number of body feathers per sample/bird with the same balance, removing the calamus before weighing to keep only the plumulaceous part of feathers. When individual feathers comprised different colour patches, we trimmed the feathers to keep only the target colour patch for which the melanin composition is known (table 1). Feather mass per sample ($M_{feathers}$) ranged between 1.2 mg (pied flycatcher) and 387.9 mg (golden eagle).

We adapted a patented procedure (p200703395 in the European Union) for the extraction of melanins from feathers [20] to remove the pigment content of the weighed feathers and thus calculate its mass contribution. Feather samples were immersed in 20% NaOH to disrupt the keratin matrix and release melanins in the solution [21], heated at 60°C for 20 min, sonicated for 10 min and vortexed for 5 s. This process was repeated, and then the samples were centrifuged at 1000*g* for 30 s to favour the sedimentation of feather particles, which were discarded. The supernatant was transferred to new tubes, which were centrifuged again to obtain a new supernatant devoid of feather particles. We treated this new supernatant with HCl to neutralize NaOH and remove sodium by inducing the reaction:


$$NaOH\;+\;HCl\;⟶\;NaCl\;+\;H_{2}O.$$

**Figure 1.**
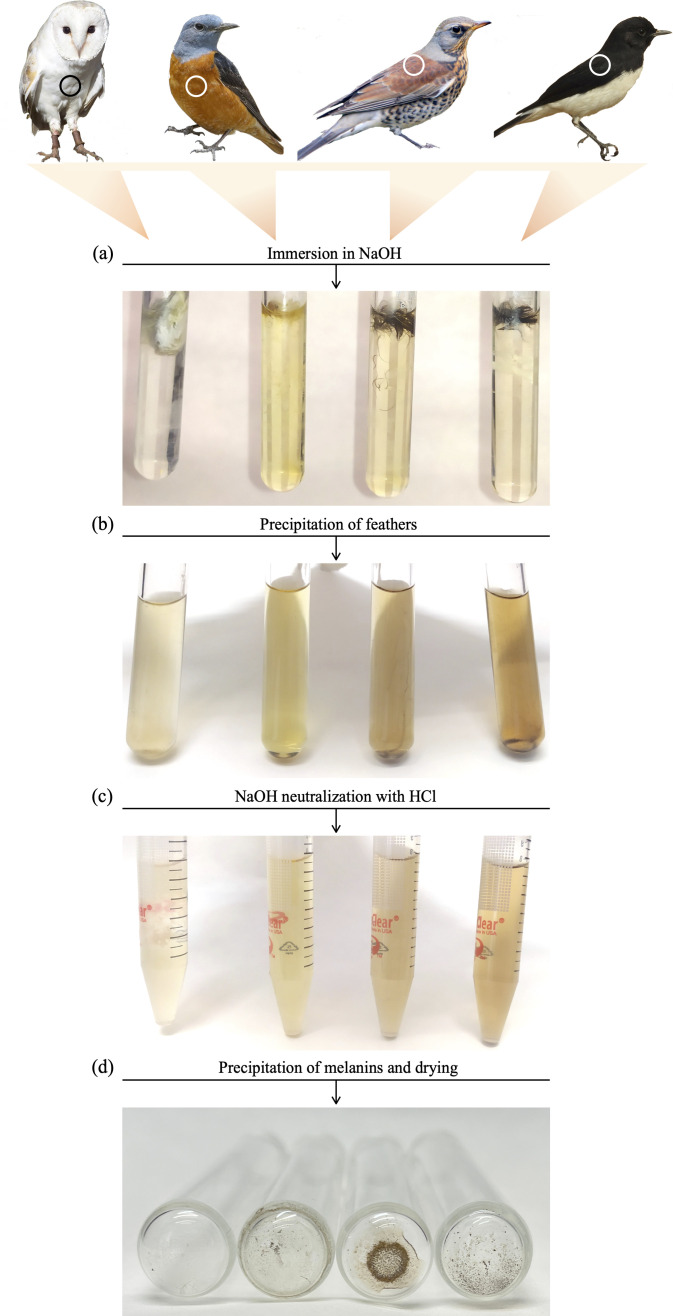
Visual representation of the main steps in the melanin extraction procedure, exemplified by feathers from four species of birds, depicted on top. From left to right: unpigmented, white barn owl *Tyto alba* feathers; pheomelanin-pigmented rufous-tailed rock thrush *Monticola saxatilis* feathers; mixed-pigmented fieldfare *Turdus pilaris* feathers; eumelanin-pigmented variable wheatear *Oenanthe picata* feathers. Circles on bird photographs indicate the plumage patches from which feathers were collected. Tubes correspond to these species in the same order as shown in the photographs. Bird photographs’ credits: barn owl: Kev747 (https://creativecommons.org/licenses/by-sa/3.0/deed.en); rufous-tailed rock thrush: Birds of Gilgit-Baltistan (https://creativecommons.org/licenses/by-sa/2.0/deed.es); fieldfare: Arnstein Rønning (https://creativecommons.org/licenses/by-sa/3.0/deed.en); variable wheatear: Dr Raju Kasambe (https://creativecommons.org/licenses/by-sa/4.0/deed.en).

We then centrifuged the samples at 3000*g* for 5 min to precipitate melanins in the medium and discarded the supernatant containing salt and water. We removed the salt by washing melanin pellets with distilled water, centrifuging again at 3000*g* for 5 min and then drying the samples at 100°C for 2 h. The melanin extraction process is visually summarized in figure 1.

We finally weighed the tubes containing dried melanin granules, and subtracted from this mass the initial mass of empty tubes to calculate the mass of melanin pigments ($M_{melanins}$). We then calculated the total percentage contribution of melanins to the mass of feathers as


$$C_{total}=\frac{M_{melanins}}{M_{feathers}}\times 100.$$

### (c) Melanin’s contribution to feather mass

To calculate the specific contribution of eumelanin ($C_{eumelanin}$) and pheomelanin ($C_{pheomelanin}$) to $M_{feathers}$ in those species with a mixed melanin composition (golden eagle and red-legged partridge), we analysed the Raman spectra available for those feathers (table 1). Specifically, we used the intensity of the Raman bands at 1380 and 2000 cm^−1^, strong predictors of eumelanin and pheomelanin concentrations, respectively, in the feathers of these species (Galván *et al*. [14,17]). We thus estimated the eumelanin : pheomelanin ratio by dividing the Raman intensity values of both bands and applied it to $C_{total}$ to get an estimation of $C_{eumelanin}$ and $C_{pheomelanin}$ in those feathers. For the species with feathers with a pure melanin composition (table 1), $C_{total}=C_{eumelanin}\;or\;C_{pheomelanin}$.

We also used the Raman spectra of pied flycatcher feathers to calculate the DHICA : DHI ratio and thus test for an association between the eumelanin subunit composition and $C_{eumelanin}$. DHICA : DHI ratio was estimated as the sum of Raman intensity in the wavenumber ranges of 550–1200 and 1650–2300 cm^−1^ in the eumelanin Raman spectra of pied flycatchers, separately for grey and black feathers [16].

### (d) Statistical analyses

We tested for differences between $C_{eumelanin}$ and $C_{pheomelanin}$ by means of analyses of variance (ANOVA) with melanin form as a fixed factor, separately analysing all samples (i.e. feathers with mixed and pure melanin composition) and samples with pure melanin composition only. We also used ANOVA to test for a difference in $C_{total}$ between feathers with mixed and pure melanin composition. Finally, we tested for correlations between $C_{eumelanin}$ and DHICA : DHI ratio in male pied flycatcher feathers using Pearson’s correlation tests. We used pied flycatcher feathers only for the latter correlation tests to avoid a strong imbalance towards eumelanin in samples with pure melanin composition, given the relatively high sample size available for pied flycatchers (table 1). As the sampling unit in our study is the feather sample and not the species, we did not apply phylogenetic corrections to the analyses.

## 3. Results

**Figure 2.**
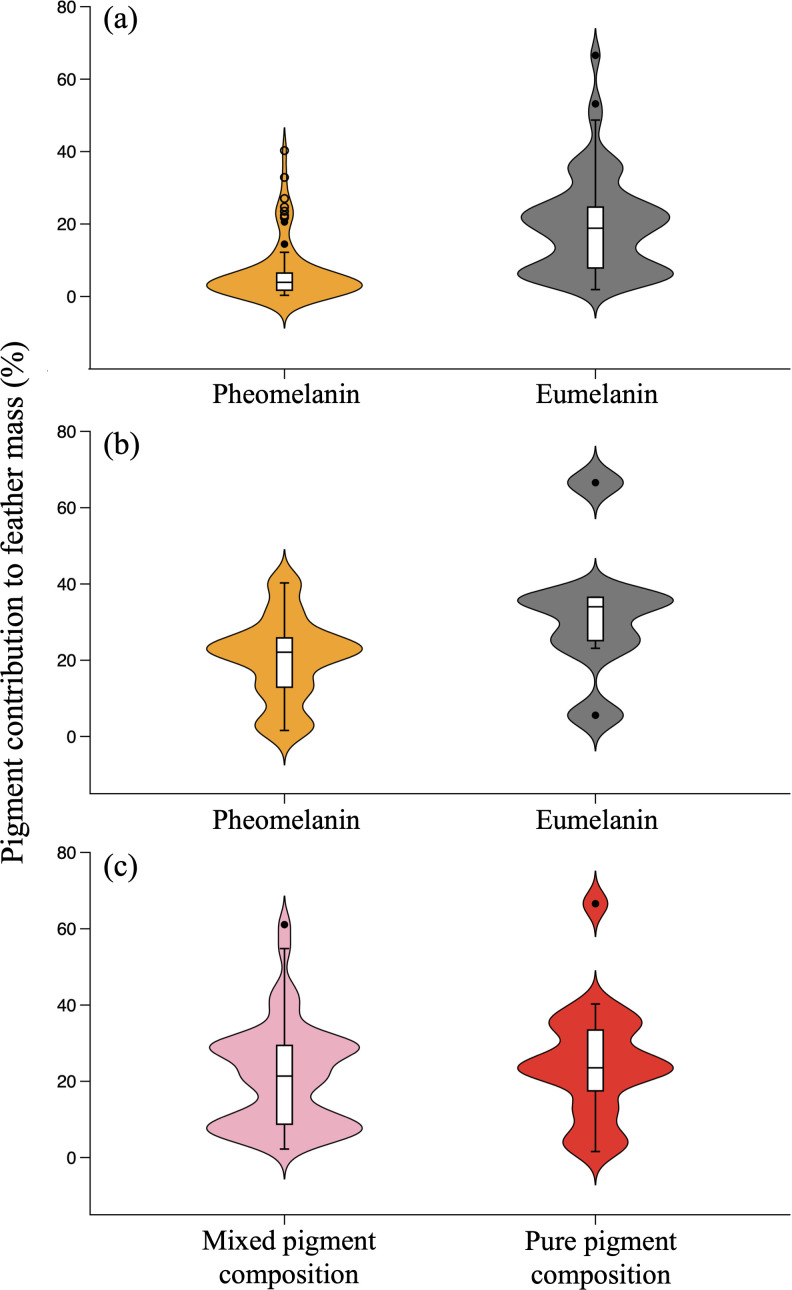
Violin plots with overlaid box plots showing the quantified contribution of melanin forms to the mass of feathers. (a) Pheomelanin and eumelanin contributions in both feathers with mixed pigment composition and feathers with pure (pheomelanin or eumelanin only) pigment composition. (b) Pheomelanin and eumelanin contributions only in feathers with pure pigment composition. (c) Total pigment contribution in feathers with mixed and pure pigment composition. Boxes are drawn from the first to the third quartile, with the median as a central line, and whiskers show the 1.5 × interquartile range (dots represent outliers).

We did not find pigment residues in the tubes with extractions from white, unpigmented barn owl feathers that we used as controls (figure 1d), which weighed the same after drying as the empty tubes, thus validating the pigment extraction method. Considering all other feather samples except those from the pied flycatcher, the mean (± s.e.) value of $C_{total}$ was 22.32% ± 1.62, ranging from 1.57% (horned grebe *Podiceps auritus*) to 66.57% (raven *Corvus corax*), with a CV of 0.62 (figure 2a; electronic supplementary material, table S1). $C_{eumelanin}$ (19.43% ± 1.72) was significantly higher than $C_{pheomelanin}$ (6.78% ± 1.04; figure 2a), with the model explaining 25% of variance in pigment contribution to $M_{feathers}$ (F_1,123_ = 41.19, *p* = 2.71 × 10^−9^). Considering only 19 samples purely composed of eumelanin or pheomelanin (table 1), $C_{eumelanin}$ values higher than $C_{pheomelanin}$ were again observed (figure 2b), although the tendency was marginally non-significant (F_1,17_ = 3.39, *p* = 0.083). In contrast, there was no difference in $C_{total}$ between feathers with mixed and pure melanin composition (F_1,70_ = 0.95, *p* = 0.334; figure 2c), with values that were similar and around 25% (mixed: 21.37% ± 1.82, pure: 24.95% ± 4.45).

**Figure 3.**
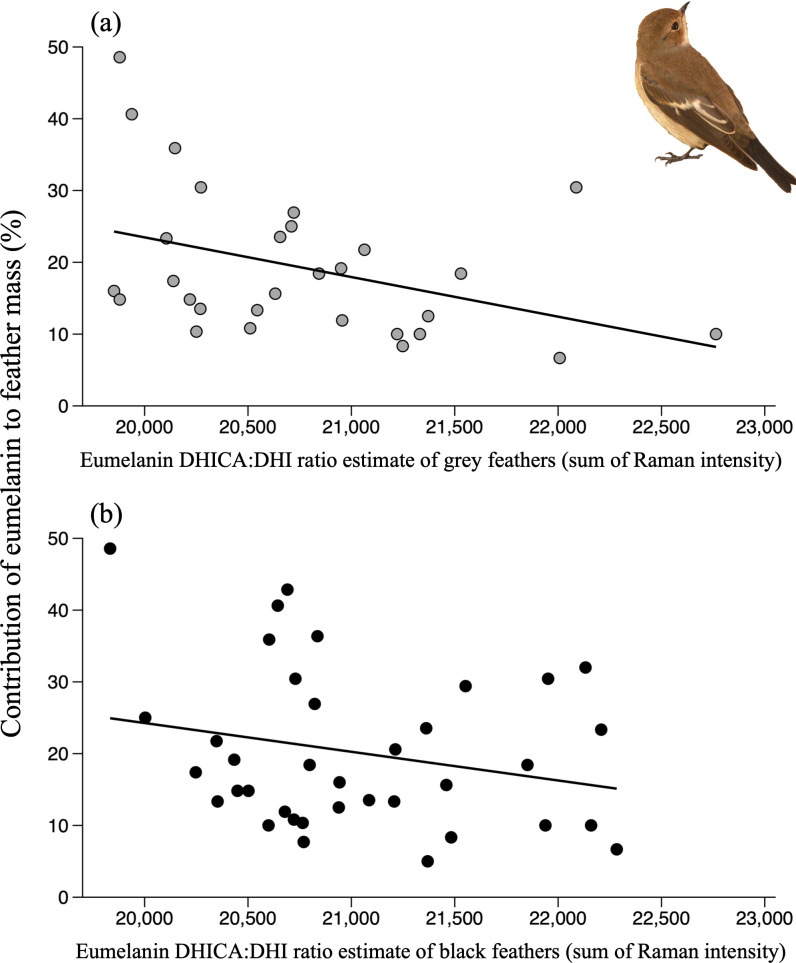
Scatterplots showing the relationship between the contribution of eumelanin to feather mass and the subunit composition of the constituent eumelanin (DHICA : DHI ratio, as estimated from Raman spectroscopy) in (a) grey feathers (*y* = 133.89 − 0.0055 *x*) and (b) black feathers from the back of adult male pied flycatchers (depicted in insert photograph; credit: Ismael Galván). Lines are the best fit lines.

Considering male pied flycatcher samples, there was a significant negative correlation between $C_{eumelanin}$ and DHICA : DHI ratio in grey back feathers (*r* = −0.39, *n* = 29, *p* = 0.035), indicating that the DHI moiety of eumelanin has a greater contribution to feather mass than the DHICA moiety (figure 3a). The same correlation, however, was not significant in the case of black feathers (*r* = −0.23, *n* = 37, *p* = 0.162; figure 3b).

## 4. Discussion

Our analyses show that the contribution of melanin pigments to the mass of feathers is highly variable, but it can reach over 60% in eumelanin-pigmented feathers such as those of ravens. The average contribution, however, is 22.3% in the bird species examined in this study. Unfortunately, this value cannot be described as low or high because there is no other quantity to which it could be relative, as plumage weight limits for flight capability have not been calculated. However, it is established that feather structures and hollow bones are adaptations that have evolved in birds because they jointly maximize flight performance with a minimum weight penalty [22,23]. In accordance with this, a review of estimates of plumage mass contribution to total body mass in 107 species of birds representing 11 orders shows this to range from 1.5 to 15.6% and averages 6.4%, with considerably lower values in ratites [24]. A very similar average value of 6.3% contribution of plumage to body mass was also estimated by Turček [25] for 91 species of birds. As a consequence, feathers are not considered to contribute significantly to overall body mass [26,27], despite being feasible to produce more and heavier feathers that provide thermoregulatory benefits [28,29]. It is thus expected that the low contribution of plumage to bird body mass responds to weight constraints due to flight requirements. If feathers must be lightweight as a response to such pressure, then our estimated $C_{total}$ value of 22.3% can be considered remarkably high, as it indicates that pigments add about a fourth part to feather mass, and then pigmented feathers add a fourth part to body mass relative to unpigmented feathers.

It is therefore likely that a lack of pigmentation allows for the allocation of more metabolic resources to the production of more or heavier feathers (i.e. containing thicker and more dense keratinous elements), without exceeding the addition of mass to the whole body that pigmented feathers would exert. This may create more insulating plumages [29,30]. This, in turn, may explain the evolution of predominantly white plumages in Arctic land birds, even in predatory species that do not rely on camouflage, such as the snowy owl *Bubo scandiacus* and the gyrfalcon *Falco rusticolus*, a question that has remained problematic [31,32]. Birds with white plumage may thus be avoiding selective pressures for pigmentation and acquiring as a consequence an ability to tolerate lower temperatures, possibly due to structural instead of optical properties of white plumage as suggested at least under high wind speeds [33].

But most animals are under selective pressures for pigmentation, and in fact, it is believed that it was the optical properties of plumage that drove the evolution of the first branching feathers [2]. Our results show that eumelanin contributes more to the mass of feathers than pheomelanin, as expected from how the different melanin forms polymerize [8,9]. As eumelanin and pheomelanin give rise to different plumage colour phenotypes, the former being mainly responsible for black, grey and dark brown colours and the latter for light brown and orange colours [6], the different colour phenotypes are associated with significantly different plumage weights. This suggests that plumages profusely pigmented by eumelanin may entail higher metabolic costs for flight than less pigmented or pheomelanin-based pigmented plumages, indeed introducing selective pressures that have been overlooked when searching for general explanations for the observed variability in bird coloration, one of the most diverse phenotypic patterns in nature [34,35]. For example, these findings may explain the results of a comparative study of wheatears (genus *Oenanthe*) showing that the evolution of migratory habits, with the flight energetic costs that this implies, is associated with species whose plumage is more profusely pigmented by pheomelanin instead of eumelanin (own unpublished data, 2025).

We also found mass differences between the subunits of eumelanin among samples of pied flycatchers, as eumelanins with lower DHICA : DHI ratios contributed more to feather mass, as expected from coupling options during DHI/DHICA polymerization [12]. Interestingly, we found such differences in the grey feathers of pied flycatchers but not in the black feathers. Grey plumage phenotypes are generated by eumelanins with a lower DHICA composition than black phenotypes, and the same can be applied to lighter grey phenotypes relative to darker grey phenotypes [6]. This finding opens an overlooked intraspecific signalling potential for species of birds that show variability in the colour intensity (i.e. reflectance) of grey feathers, such as the pied flycatcher [16], as displaying lighter grey feathers may entail higher metabolic costs.

Our findings also show that feathers with mixed and pure melanin composition contribute the same to feather mass, and this contribution can be established at approximately 25%. This suggests that melanin forms do not exert an additive effect on feather mass. Instead, there may be a limit for feather pigmentation. Such a limit might arise during the genetic control of melanin deposition in the growing feather follicles [5], and might be under selection, likely associated with other selective pressures for flight efficiency such as those leading to small body size [36].

In sum, our study shows that melanins, the most common pigments in birds, make a significant contribution to the mass of feathers. As plumage must be lightweight to facilitate flight, the mass contribution of melanins is likely to be involved in evolutionary trade-offs between the benefits of pigmentation and metabolic costs derived from increased weight. Such costs differ between the two main forms of melanin, as eumelanin appears to be heavier than pheomelanin, and also between the subunits of eumelanin. Future studies should investigate the evolutionary implications of these differences in melanin mass for the distinct colour phenotypes generated by different melanin forms, as well as the contribution to feather mass of other pigment classes such as carotenoids. Our established limit for feather pigmentation at approximately 25% of feather mass should also be viewed as a potential evolutionary constraint for the adaptive benefits of pigmentation and should be investigated in such a context.

## Data Availability

The datasets supporting this article are provided in the electronic supplementary material, table S1. Supplementary material is available online [37].

## References

[1] Terrill RS, Shultz AJ. 2023 Feather function and the evolution of birds. Biol. Rev. **98**, 540–566. (10.1111/brv.12918) 36424880

[2] Persons WS, Currie PJ. 2019 Feather evolution exemplifies sexually selected bridges across the adaptive landscape. Evolution **73**, 1686–1694. (10.1111/evo.13795) 31359437

[3] Galván I, Solano F. 2016 Bird integumentary melanins: biosynthesis, forms, function and evolution. Int. J. Mol. Sci. **17**, 520. (10.3390/ijms17040520) 27070583 PMC4848976

[4] Lingham-Soliar T. 2014 Feather structure, biomechanics and biomimetics: the incredible lightness of being. J. Ornithol. **155**, 323–336. (10.1007/s10336-013-1038-0)

[5] Lin SJ *et al*. 2013 Topology of feather melanocyte progenitor niche allows complex pigment patterns to emerge. Science **340**, 1442–1445. (10.1126/science.1230374) 23618762 PMC4144997

[6] Galván I, Wakamatsu K. 2016 Color measurement of the animal integument predicts the content of specific melanin forms. RSC Adv. **6**, 79135–79142. (10.1039/c6ra17463a)

[7] Ito S, Wakamatsu K, d’ischia M, Napolitano A, Pezzella A. 2011 Structure of melanins. In Melanins and melanosomes: biosynthesis, biogenesis, physiological, and pathological functions (eds J Borovanský, Riley PA), pp. 167–185. Weinheim, Germany: Wiley-Blackwell. (10.1002/9783527636150)

[8] Piletic IR, Matthews TE, Warren WS. 2009 Estimation of molar absorptivities and pigment sizes for eumelanin and pheomelanin using femtosecond transient absorption spectroscopy. J. Chem. Phys. **131**, 181106. (10.1063/1.3265861) 19916591 PMC4108625

[9] Brumbaugh JA. 1968 Ultrastructural differences between forming eumelanin and pheomelanin as revealed by the pink-eye mutation in the fowl. Dev. Biol. **18**, 375–390. (10.1016/0012-1606(68)90047-x) 5719204

[10] Aroca P, Solano F, Salina C, García‐borrón JC, Lozano JA. 1992 Regulation of the final phase of mammalian melanogenesis. Eur. J. Biochem. **208**, 155–163. (10.1111/j.1432-1033.1992.tb17169.x) 1511683

[11] Ito S, Wakamatsu K. 2008 Chemistry of mixed melanogenesis—pivotal roles of dopaquinone. Photochem. Photobiol. **84**, 582–592. (10.1111/j.1751-1097.2007.00238.x) 18435614

[12] Panzella L, Gentile G, D’Errico G, Della Vecchia NF, Errico ME, Napolitano A, Carfagna C, d’Ischia M. 2013 Atypical structural and π‐electron features of a melanin polymer that lead to superior free‐radical‐scavenging properties. Angew. Chem. Int. Ed. **52**, 12684–12687. (10.1002/anie.201305747) 24123614

[13] Lee E, Tanaka H, Wakamatsu K, Sugita S. 2009 Melanin-based iridescent feather color in the jungle crow. J. Vet. Med. Sci. **71**, 1261–1263. (10.1292/jvms.71.1261) 19801912

[14] Galván I, Jorge A, Ito K, Tabuchi K, Solano F, Wakamatsu K. 2013 Raman spectroscopy as a non‐invasive technique for the quantification of melanins in feathers and hairs. Pigment Cell Melanoma Res. **26**, 917–923. (10.1111/pcmr.12140) 23890020

[15] Galván I *et al*. 2018 Solar and terrestrial radiations explain continental-scale variation in bird pigmentation. Oecologia **188**, 683–693. (10.1007/s00442-018-4238-8) 30094635

[16] Galván I. 2022 Pigment molecular composition reveals significant information for visual communication. Funct. Ecol. **36**, 2756–2762. (10.1111/1365-2435.14176)

[17] Galván I, Cerezo J, Jorge A, Wakamatsu K. 2018 Molecular vibration as a novel explanatory mechanism for the expression of animal colouration. Integr. Biol. **10**, 464–473. (10.1039/c8ib00100f) 29951656

[18] Rodríguez-Martínez S, Arnalds Ó, Guðmundsson J, Svavarsdóttir M, Gísladóttir FÓ, Nielsen ÓK, Galván I. 2023 Exposure to sulfur in soil explains pigmentation by pheomelanin in birds inhabiting Iceland. J. Ornithol. **164**, 639–649. (10.1007/s10336-023-02051-1)

[19] Galván I, Rodríguez‐Martínez S. 2018 Females mate with males with diminished pheomelanin‐based coloration in the Eurasian nuthatch Sitta europaea. J. Avian Biol. **49**, e01854. (10.1111/jav.01854)

[20] Galván I, Bijlsma RG, Negro JJ, Jarén M, Garrido‐Fernández J. 2010 Environmental constraints for plumage melanization in the northern goshawk Accipiter gentilis. J. Avian Biol. **41**, 523–531. (10.1111/j.1600-048x.2010.04998.x)

[21] d’Ischia M *et al*. 2013 Melanins and melanogenesis: methods, standards, protocols. Pigment Cell Melanoma Res. **26**, 616–633. (10.1111/pcmr.12121) 23710556

[22] Dumont ER. 2010 Bone density and the lightweight skeletons of birds. Proc. R. Soc. B **277**, 2193–2198. (10.1098/rspb.2010.0117) PMC2880151 20236981

[23] Sullivan TN, Pissarenko A, Herrera SA, Kisailus D, Lubarda VA, Meyers MA. 2016 A lightweight, biological structure with tailored stiffness: the feather vane. Acta Biomater. **41**, 27–39. (10.1016/j.actbio.2016.05.022) 27184403

[24] Brassey CA, Sellers WI. 2014 Scaling of convex hull volume to body mass in modern primates, non-primate mammals and birds. PLoS One **9**, e91691. (10.1371/journal.pone.0091691) 24618736 PMC3950251

[25] Turček FJ. 1966 On plumage quantity in birds. Ekol. Pol. Ser. **14**, 617–634.

[26] Atkins-Weltman K, Snively E, O’Connor P. 2021 Constraining the body mass range of Anzu wyliei using volumetric and extant-scaling methods. Vertebr. Anat. Morphol. Palaeontol. **9** 95-104. (10.18435/vamp29375)

[27] Larramendi A, Paul GS, Hsu SY. 2021 A review and reappraisal of the specific gravities of present and past multicellular organisms, with an emphasis on tetrapods. Anat. Rec. **304**, 1833–1888. (10.1002/ar.24574) 33258532

[28] De La Hera I, Pérez‐Tris J, Tellería JL. 2010 Relationships among timing of moult, moult duration and feather mass in long‐distance migratory passerines. J. Avian Biol. **41**, 609–614. (10.1111/j.1600-048x.2010.05075.x)

[29] Nord A, Holje V, Judik B, Folkow LP, Pap PL. 2023 Seasonal changes in plumage density, plumage mass, and feather morphology in the world’s northernmost land bird, the Svalbard rock ptarmigan (Lagopus muta hyperborea). Polar Biol. **46**, 277–290. (10.1007/s00300-023-03118-8)

[30] Dyck J. 1979 Winter plumage of the rock ptarmigan: structure of the air-filled barbules and function of the white colour. Dan. Ornitol. Foren. Tidsskr. **73**, 41–58.

[31] Tickell WLN. 2003 White plumage. Waterbirds **26**, 1–12. (10.1675/1524-4695(2003)026[0001:WP]2.0.CO;2)

[32] Bortolotti GR. 2006 Natural selection and coloration: protection, concealment, advertisement, or deception? In Bird coloration, vol. 2: function and evolution (eds GE Hill, KJ McGraw), pp. 3–35. Cambridge, MA: Harvard University Press. (10.2307/j.ctv22jnr8k.4)

[33] Walsberg GE, Campbell GS, King JR. 1978 Animal coat color and radiative heat gain: a re-evaluation. J. Comp. Physiol. B **126**, 211–222. (10.1007/BF00688930)

[34] Cooney CR, He Y, Varley ZK, Nouri LO, Moody CJA, Jardine MD, Liker A, Székely T, Thomas GH. 2022 Latitudinal gradients in avian colourfulness. Nat. Ecol. Evol. **6**, 622–629. (10.1038/s41559-022-01714-1) 35379937

[35] Delhey K, Valcu M, Muck C, Dale J, Kempenaers B. 2023 Evolutionary predictors of the specific colors of birds. Proc. Natl Acad. Sci. USA **120**, e2217692120. (10.1073/pnas.2217692120) 37579151 PMC10450850

[36] Hone DWE, Dyke GJ, Haden M, Benton MJ. 2008 Body size evolution in Mesozoic birds. J. Evol. Biol. **21**, 618–624. (10.1111/j.1420-9101.2007.01483.x) 18194232

[37] Galván I, Gómez-Vicioso J. 2025 Supplementary material from: Pigment contribution to feather mass depends on melanin form and is restricted to approximately 25%. Figshare. (10.6084/m9.figshare.c.7953924)40829652

